# V–V ECMO for severe *Chlamydia psittaci* pneumonia presenting with sudden cardiac arrest: A case report and literature review

**DOI:** 10.1097/MD.0000000000039808

**Published:** 2024-11-08

**Authors:** Juan Chen, Yong Sun, Jian Luo, Yang Wu, Kaiyu Wang, Weiwen Zhang, Honglong Fang

**Affiliations:** a Department of Clinical Laboratory, The Quzhou Affiliated Hospital of Wenzhou Medical University, Quzhou People’s Hospital, Quzhou, Zhejiang, China; b Zhejiang University of Traditional Chinese Medicine, Hangzhou, Zhejiang, China; c Department of Critical Care Medicine, The Quzhou Affiliated Hospital of Wenzhou Medical University, Quzhou People’s Hospital, Quzhou, Zhejiang, China.

**Keywords:** cardiac arrest, *Chlamydia psittaci*, mNGS, psittacosis, V–V ECMO

## Abstract

**Rationale:**

Psittacosis, also known as parrot fever, is an infectious disease caused by *Chlamydia psittaci*, which can lead to *C psittaci* pneumonia. Clinical manifestations are highly nonspecific, which can vary from asymptomatic infection to severe pneumonia and even death.

**Patient concerns:**

In this case presentation, we reported one 65-year-old male case of *C psittaci* pneumonia who was admitted to our hospital on December 2, 2022 due to the chief complaints of poor appetite and fatigue for 3 days as the clinical manifestations. He denied contact with birds but admitted riding horses 1 week ago.

**Diagnoses:**

Chlamydia psittaci pneumonia of patient was confirmed through metagenomic sequencing of bronchoalveolar lavage fluid under bronchoscopy.

**Intervention:**

Patient was treated with V-V ECMO, invasive mechanical ventilation and CRRT.

**Outcomes:**

On December 12, the patient was successfully weaned off V–V ECMO and discharged on December 20, 2022. During postoperative follow-up, CT scan in a local hospital revealed the infiltrative lesions of the lung were absent.

**Lessons:**

This case prompts that metagenomic next-generation sequencing is a feasible diagnostic tool for psittacosis, which can rapidly worsen and even cause sudden cardiac arrest. V–V ECMO might be a viable emergency therapeutic option.

## 1. Introduction

Psittacosis, which is also known as parrot fever, is an infectious disease caused by *Chlamydia psittaci*. Clinical manifestations are highly nonspecific, which can vary from asumptomatic infection to severe pneumonia, acute respiratory distress syndrome and even multiple organ failure.^[1,2]^ With the widespread application of metagenomic next-generation sequencing (mNGS) in the diagnosis of infectious diseases, the prevalence of *C psittaci* pneumonia has been gradually increasing year by year in clinical settings. The typical clinical manifestations of *C psittaci* pneumonia include high fever, chills, dry cough, muscle aches, and dyspnea, etc. In addition to these typical clinical manifestations, a low proportion of patients may present with digestive system symptoms, such as diarrhea, as well as nervous system symptoms, such as speech disorder, as the first symptoms.^[2–5]^ In this case report, we described the successful mNGS-based diagnosis and vein–vein extracorporeal membrane oxygenation (V–V ECMO) treatment of a male patient with *C psittaci* pneumonia who presented with sudden cardiac arrest admitted to Quzhou People’s Hospital as follows.

## 2. Case presentation

Participant have provided their written informed consent to participate in this study.

**Figure 1.**
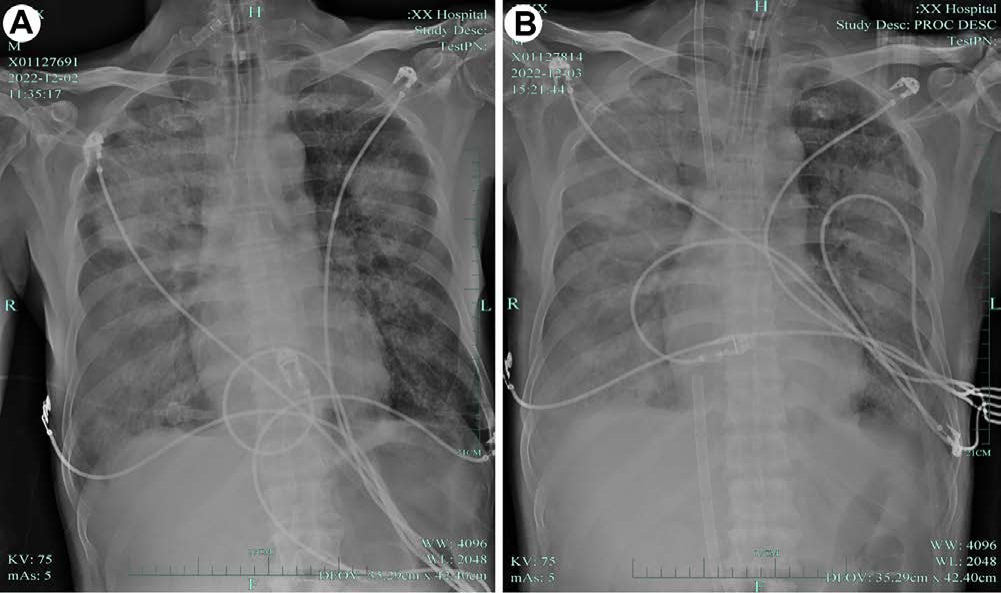
Bedside chest X-ray on December 2, 2022 (A) and December 3, 2022 (B).

On December 2, 2022, the 65-year-old male patient was admitted to our hospital due to poor appetite and fatigue for 3 days and unconsciousness for 2 hours. During the waiting period in Department of Gastroenterology, the patient suddenly became unconscious, and the carotid pulse was absent. He was immediately given with continuous chest compression and transferred to ICU. He was delivered with intravenous injection of 1 mg of epinephrine, tracheal intubation, and respiratory mechanical ventilation, etc. After approximately 12 minutes, sinus rhythm was restored and he was transferred to our department for further treatment. Physical examination: the patient was in coma, ventilator-assisted breathing through tracheal intubation (pressure-control mandatory ventilation mode, pressure control 18 cmH_2_O, positive end-expiratory pressure [PEEP] 10 cmH_2_O, fraction of inspiration O_2_ 1.0), body temperature 35.8 °C, pulse rate 135 times/min, blood pressure 118/67 mm Hg maintained by 0.4 μg/kg of noradrenaline. He had slow light reflex, asymmetrical breathing sounds and audible moist rales in both lungs, especially significant on right side, regular heart rhythm, no pathological murmur in all heart valves, soft abdomen, tenderness and rebound pain in the whole abdomen, liver and spleen were umpalpable under the ribs, no bowel sounds, unable to cooperate with limb muscle strength examination, cold extremities, and negative bilateral Babinski sign. Blood gas analysis revealed pH 6.93, carbon dioxide partial pressure: 77.2 mm Hg, oxygen partial pressure: 56.8 mm Hg, bicarbonate concentration: 10.0 mmo1/L, alkali residue: −27.3 mmo1/L, potassium: 4.67 mmo1/L, lactic acid: 12.1 mmo1/L. Laboratory examination: alanine aminotransferase 108.5 U/L, creatinine 139.3 μmo1/L, creatine kinase 211.0 U/L, lactate dehydrogenase 367.1 U/L, creatine kinase isoenzyme 21.0 U/L, high-sensitivity troponin T 0.334 μg/L, brain natriuretic peptide 1719 pg/mL and high-sensitivity C-reactive protein 233.35 mg/L, procalcitonin 34.31 ng/mL. Routine blood test: white blood cell count 11.5 × 10^9^/L, neutrophil% 88.0%, absolute value of lymphocyte 0.33 × 10^9^/L, hemoglobin 125 g/L, and platelet 85 × 10^9^/L. Bedside color Doppler echocardiography: the motion amplitude of left ventricular wall was decreased slightly (ejection fraction 48%), a small amount of mitral and tricuspid valve regurgitation and tachycardia; bedside B-ultrasound of abdomen detected a left renal cyst. B-ultrasound showed a small amount of pleural effusion on the right side. Electrocardiogram revealed sinus tachycardia and T wave changes. Bedside chest X-ray found multiple infiltration foci in both lungs, especially significant in the right lung. The possibility of pulmonary edema was considered (Fig. 1A). Admission diagnosis: the cause of cardiac arrest remained to be identified. The possibilities of cardiogenic, pulmonary embolism, or severe pneumonia were considered. Besides, metabolic acidosis and multiple organ failure were diagnosed. Noradrenaline was given to elevate blood pressure, continuous renal replacement therapy (CRRT) was adopted for dehydration and microenvironment adjustment, ventilator-assisted breathing was used to improve oxygenation, and 0.5 g imipenem was given intravenously for q6h as anti-infection therapy. After dehydration and ventilator adjustment, the patient still showed severe hypoxemia with an oxygenation index of 68.5. Therefore, V–V ECMO was performed at a rotation speed of 3000 r/min, flow rate of 3.5 L/min, oxygen flow rate of 3.5 L/min and oxygen concentration of 1.0. After ECMO was established, protective lung ventilation strategy was adopted (pressure-control mandatory ventilation mode, pressure control 13 cmH_2_O, PEEP 10 cmH_2_O, f 10 times/min, fraction of inspiration O_2_ 0.5).

**Figure 2.**
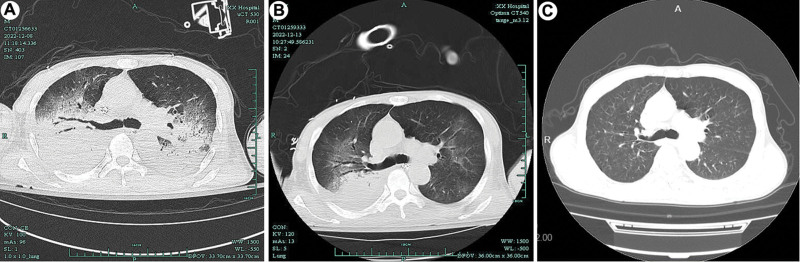
Chest CT scan on December 8, 2022 (A), December 12, 2022 (B) and January 10, 2023 (C).

**Figure 3.**
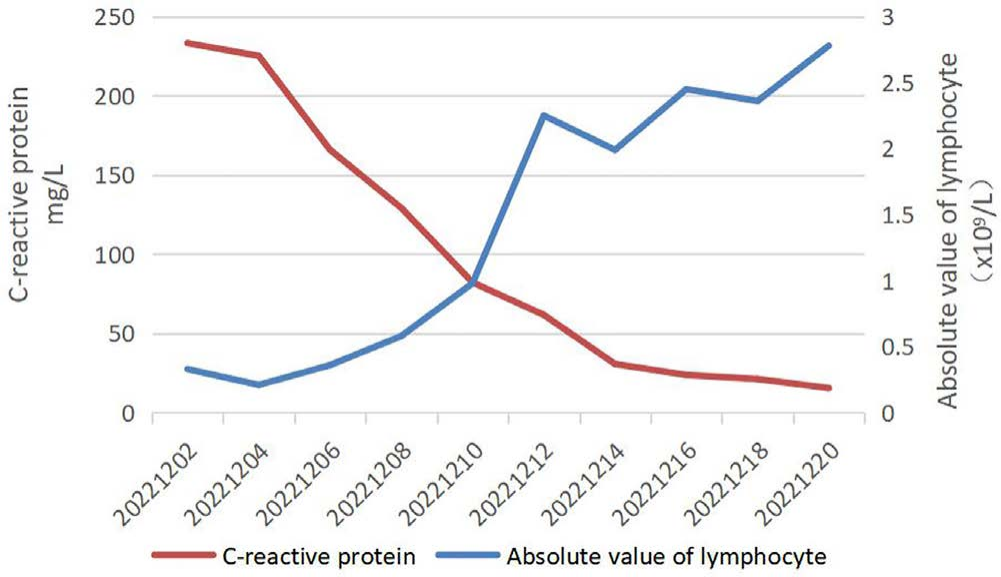
Changing patterns of C-reactive protein and absolute value of lymphocyte.

Metagenomic next-generation sequencing of bronchoalveolar lavage fluid (BALF) and microbial culture was performed. After 24-h treatment, the dose of noradrenaline was not changed, and blood lactic acid level was not significantly decreased. Chest X-ray showed more infiltrative lesions in the lungs (Fig. 1B). On December 4, mNGS indicated *C psittaci* (RPTM 147685), and the diagnosis of severe pneumonia caused by *C psittaci* was confirmed. The anti-infection regimen was adjusted to omadacycline combined with doxycycline. On December 7, the patient’s lactic acid level returned to normal, the absolute value of blood lymphocytes began to rise, and C-reactive protein (CRP) value and noradrenaline dose were gradually decreased. CT scan of the chest revealed multiple infection foci in bilateral lungs, a slight amount of pleural effusion on both sides with partial pulmonary tissue atelectasis (Fig. 2A). At 1 week after adjusting the anti-infection regimen, the patient’s blood pressure and urine volume returned to normal. After discontinuing use of sedatives, his consciousness became clear, CRP value was decreased significantly, the absolute value of lymphocytes was normal (Fig. 3). V–V ECMO was weaned off and CRRT was discontinued on December 12, 2022. Chest CT scan on December 13, 2022 showed that the quantity of infiltrative foci in both lungs was significantly less compared with that on December 7 (Fig. 2B). On December 14, tracheal intubation was removed, and antibiotics use was adjusted to doxycycline alone. On December 20, the patient was discharged, and doxycycline was administered for 3 weeks. On January 10, 2023, chest CT scan detected no infiltrative lesions in bilateral lungs in a local hospital (Fig. 2C).

## 3. Discussion

*Chlamydia psittaci* is a Gram-negative aerobic intracellular parasitic pathogenic microorganism, which primarily lives in birds or poultry, such as parrots, pigeons, chicken, crows, and geese, etc. Human body is mainly infected by inhaling or contacting the aerosol containing *C psittaci*.^[6,7]^ Previously, it is highly difficult to diagnose *C psittaci* pneumonia due to limited clinical testing approaches.^[8]^ In recent years, with the widespread application of mNGS technology in infectious diseases, clinical understanding of this disease has been deepened. At present, it is considered that most patients with psittacosis have a clear history of contact with birds or poultry. After infection, respiratory symptoms, such as high fever, cough, and dyspnea, can occur in infected individuals,^[9,10]^ and a minority of patients may develop extrapulmonary manifestations, such as digestive tract and nervous system disorders.^[4,5]^

In this case, the sequence number of *C psittaci* in BALF was extremely high, but the patient denied any clear history of contact with birds or poultry. After explicit inquiry, he had a history of “riding horses” 1 week ago. Studies have confirmed that horses are the source of infection of *C psittaci*.^[11,12]^ It is widely recognized that *C psittaci* is mainly identified in male patients aged 50 to 65 years. Laboratory examination showed normal or slightly increased white blood cell count, significantly increased amount of CRP in this case, which are consistent with previous findings.^[9,13,14]^ In the early stage, the absolute number of peripheral lymphocytes of this patient was continuously lower than the normal value, which gradually returned to normal with the improvement of this disease, suggesting that *C psittaci* might damage the immune function. Previous studies have indicated that *C psittaci* infection would lead to immune disorder after activating the innate immune system, which eventually provoking increased lymphocyte apoptosis and immunosuppression.^[15,16]^ Different from other cases, this patient’s initial symptoms are not typical without fever, cough or dyspnea, and his condition progresses rapidly.

After successful cardiopulmonary resuscitation, he still experienced hypotension and severe dyspnea. Emergency cardiac color Doppler ultrasound showed that the heart size was normal and the left ventricular systolic function was slightly weakened with an ejection fraction of 48%. Bedside chest X-ray showed multiple infiltrative foci in both lungs, and the possibility of pulmonary edema was considered. Blood pressure was maintained by 0.4 μg/kg noradrenaline. Severe hypoxemia was the prominent manifestation. CRRT dehydration and increasing PEEP yielded poor efficacy for pulmonary edema. After 2 hours, the oxygenation index was <80, and then V–V ECMO was adopted to improve the oxygen supply. Severe hypoxemia in this case was not simply caused by pulmonary edema. Chest X-ray indicated evident exudative lesions of the right lung. Combined with significantly increased CRP, the risk of lung infection should be considered. Imipenem was given for anti-infection treatment, and mNGS of BALF was performed to confirm the diagnosis.

According to literature review, *C psittaci* pneumonia with hidden early symptoms and rapidly progressing into cardiac arrest has been rarely reported. The cause of cardiac arrest remains elusive. His family members memorized that the patient’s lips were purple after fatigue and poor appetite for 1 day. Besides, he presented with refractory hypoxemia after successful cardiopulmonary resuscitation. We speculate that the possibility of silent hypoxia should be considered in this case, which is characterized by the presence of hypoxia without dyspnea and first proposed in COVID-19 patients, and its specific mechanism is poorly understood.^[17]^

*Chlamydia psittaci* can enter the human body, proliferate in endothelial cells and then spreads to various organs of the human body through blood flow, thereby affecting the liver, kidney and heart, and even resulting in multiple organ dysfunction,^[7,10]^ which is evidenced by mNGS by Xu et al.^[9]^ However, in this case, mNGS was not performed in peripheral blood, and there was no definite evidence that the patient’s multiple organ dysfunction was directly caused by *C psittaci*, which may be directly correlated with hypotension and severe hypoxemia after cardiopulmonary resuscitation. After active treatment, all organ functions were properly recovered in this case. This patient rapidly progressed from fatigue into cardiac arrest, and positive symptoms and signs were quite limited, which brought significant challenges to clinical diagnosis. Use of V–V ECMO saved time for further diagnosis and treatment. In addition, mNGS also played a critical role in confirming the diagnosis.

## 4. Conclusion

Taken together, prompt diagnosis and efficacious interventions should be delivered for patients with *C psittaci* pneumonia. More importantly, extensive attention should be diverted to those patients with mild clinical symptoms during the initial stage of onset. Otherwise, it may rapidly progress or aggravate into critical diseases or even death. Nevertheless, long-term follow-up remains to be carried out.

## Author contributions

**Conception and Writing of Manuscript:** Juan Chen.

**Patient Management and Data Collection:** Yong Sun, Jian Luo, Yang Wu, Kaiyu Wang, Weiwen Zhang.

**Review and Revision of Manuscript:** Fang Honglong.
